# Can Pyorrhea Alveolaris Be Cured?

**Published:** 1898-06

**Authors:** J. M. Thompson


THE DENTAL REGISTER.

VOL. LII.] JUNE, 1898. [No. 6.

### COMMUNICATIONS.

### Can Pyorrhea Alveolaris Be Cured?

BY J. M. THOMPSON, D.D.S.

Read before the Detroit Dental Society.

In bringing such a question before the society, it is my purpose to appeal to each member in such a way that all may be interested, and with the desire that an answer in the affirmative may be one of its immediate results.

The general use of the term Pyorrhea Alveolaris has given to many a somewhat vague idea of a group of troubles which only those of our profession who have worked and studied without ceasing have been able to classify. You must thank, for your paper this evening, such authorities as Black, Barret, Pierce, and Burchard; also some others whom I shall quote later.

The presence of pus in any organ of the body indicates the presence of an irritant, and we can not have continued irritation without inflammation and pyogenic conditions as a result. A piece of wooden toothpick may as a simple irritant cause an abscess beneath the alveolar border. This would be a simple case, but would be Pyorrhea Alveolaris just the same. The use of the term in the dental profession seems closely related to malaria as used by our medical brethren. We are not here to study terminology, so I will proceed with our subject.

To be able to recognize the abnormal we must first know what is normal, and I will read Dr. Black's description of a normal gum and surrounding tissues: "The exposed surface of the free margin of the gum is covered with a very dense and squamous epithelium which fits it well to withstand the severe abraiding contact with food necessary in the act of mastication. This rests upon a layer of cells which cover a series of papillae projected from the fibrous tissue beneath as a glove covers the fingers; the whole when in normal condition rests on the rim of the alveolus and is drawn snugly around the neck of the tooth, forming a strong resistant, yet flexible cushion, to the tissues which it protects. It is strongly attached to the neck of the tooth and periosteum of the wall of the alveolus by a radiating bundle of fibrous tissue, which have become known as the dental ligament. That part of the membrane which lies next to the tooth is of different structure than its other parts. Here it is clothed with a very soft round or polygonal gland like epithelium that suggests the formation of a gland, but fails to assume glandular structure. This is called the gingival organ. This organ emits a profusion of small mucus cells termed mucus corpuscles, which are always found in the saliva."

Now that we have had the normal gum described by one of the best authorities, let us consider the different causes which may destroy the health of this tissue and those connected with it. One of the first causes which may produce an unhealthy condition of the gums, of course, is uncleanliness. From lack of care the action of the glandular membrane just described may be checked or the excretions kept from passing away into their proper channels, and these in time may become thickened and deposited upon the surface of the tooth. Sometimes, by pressing the gum, these thickened cells will exude and are deceiving in their appearance, being in many cases mistaken for pus. Salivary calculus is another evil with which we have to battle, and yet of itself it is not a stubborn irritant. True, it causes the loss of many teeth, and we are called upon every day to remove more or less of it, as the case may require, but we generally find allied with it a deposit of an entirely different character. This unseen foe is known to us as a serumal calculus. In character it is a dark, horny scale, and may be deposited upon the surface of the root without regard to position. Wherever this deposit is found there is always a destruction of the membranes, and a general breaking down is sooner or latter accomplished.

Dr. W. C. Barrett describes three distinct classes of pyorrhea, viz: gingival pyorrhea, nodular pyorrhea, and cachetic pyorrhea. Dr. Black's theory regarding infection is not without foundation, but I am of the opinion that Dr. Barrett is right when he says that the nodular deposit is the initial lesion, and all troubles arise from an accumulation of nodules. Dr. Black substantiates his claims by the fact that the process seems to be the part most affected and that the absorption leaves the edges rounded instead of ragged, and also that they become thickened. This condition of the alveolar process is a great hindrance to the treatment of pyorrhea, and although he gives some outlines of radical methods, he does not give any definite and positive assurance of a cure.

The etiology of gingival pyorrhea as given by Dr. Barrett is very clear and it reads thus: "The etiology, while it may rest in a general atony, is not cachectic, but rather accidental. It begins in follicular stomatitis, which produces an altered condition of follicles and of their secretion, and this of itself becomes the local irritant which intensifies the state."

The pathology consists in the morbid changes in the follicles and the hyperaemic condition of the gums, with a great degree of edema and infiltration of the soft tissues; a pouring out of plastic exudate and its infection, and breaking down all these in turn, by contiguity of its tissue and by local irritation, producing an inflammation of the pericemental membrane with osteitis and wasting edges of the alveolar walls. It is this condition which is so frequently mistaken for a worse one, and, in my opinion, this is the class of disturbances, cures of which are so often related in journals and at dental meetings, as the result of a few days of empirical treatment." He also goes on to say that the prognosis in such cases is decidedly favorable. For his theories upon nodular and cachectic pyorrhea. I would refer you to page 526, *Dental Cosmos*, Vol. 35 of 1893.

Dr. Burchard, in the January number of the *Cosmos* of this year, describes five distinct varieties of dental calculi. He claims that they are distinct in that each is associated with distinctive pathological states and chemical conditions. Three of these classes (two of them undoubtedly) are of salivary origin, the doubtful member of at least partial salivary origin, while two of them may be fitly termed seminal or hematogenic. Two or more varieties may exist upon the same case at the same time. He further states that the presence of these deposits excites progressive degenerative changes resulting in the destruction of the process and pericimentum. His fourth variety is called a variety because its appearance is always associated with long continued suppuration and seems a consequence of such conditions. This variety is a distinctive feature of the class of pyorrhea described by Dr. Black as phadegenic pericementitis, but play no necessary part in either the indication or contra-indication of the disease, and must be regarded as an epiphenomenon. Phagedænic pericementitis may be confined to one tooth or even one root of a tooth, and this fact is a diagnostic feature not found in the other varieties of pyorrhea.

Dr. Robert Eugene Payne, of New York, read a paper before the Southern Dental Association last August, and it was published in the December, '97, number, and the discussion is taken up in the January and February numbers of this year. He advocates replantation as a cure for pyorrhea, and some of the arguments for and against I am sure must have interested those who have read them. Dr. C. N. Pierce was brought into the discussion, and as we all know his theories, it is needless to consume time with them. His uric acid theories seem to me to be of great value, and is one of the few ways in which this question may be answered.

Dr. J. S. Marshall suggests that the teeth are subject to the same conditions which affect the hair at certain ages, *i. e.*, the same influences which act upon the hair bulbs may be closely related to those which act upon the membrane about the teeth.

Many other authorities might be quoted upon this subject, but time and space do not warrant further efforts. The writer firmly believes that failure of the operator to recognize the true condition of each case and to apply the correct treatment results in the loss of many teeth; also, that there are certain classes of individuals in which pyorrhea alveolaris can not be cured and is a temperamental characteristic. It may be held in abeyance, but its presence is as inevitable as the eventual death of the body.

In summing up the question, we find several distinct varieties of pyorrhea alveolaris described, and each one has its own peculiarities. The conclusions drawn are, first, that success requires an accurate diagnosis and treatment; secondly, that some varieties are almost incurable, and thirdly, the prognosis is generally unfavorable, or, as Prof. J. Foster Flagg gives it, "Possible amelioration of greater or less length of duration; probable recurrence of trouble, with usually gradual but persistent reestablishment of previous condition."

